# Creutzfeldt-Jakob Disease as a Cause of Cognitive Decline and Seizures in the Elderly: Diagnostic Pointers and Strategy for Investigation

**DOI:** 10.1155/2011/719583

**Published:** 2011-12-13

**Authors:** R. Williams, F. Cresswell, M. McClure, R. Lane

**Affiliations:** ^1^Department of Radiology, St George's Hospital, Blackshaw Road, London SW17 0QT, UK; ^2^Lister Unit Infectious Diseases Department, Northwick Park Hospital, Harrow Road, London HA1 3UJ, UK; ^3^Department of Renal Medicine, Hammersmith Hospital, Du Cane Road, London W12 0HS, UK; ^4^Department of Neurology, Charing Cross Hospital, Fulham Palace Road, London W6 8RF, UK

## Abstract

Cognitive decline affects one in twenty people over the age of 65. There is often a paucity of clues as to the underlying pathology, and while the diagnosis will usually prove to be either Alzheimer's disease or vascular dementia, there may be clinical features suggesting rarer alternatives. This case of a 71-year-old lady with a 3-month history of progressive cognitive decline illustrates clinical features suggestive of Creutzfeltd-Jakob disease such as rapid decline in conscious level and myoclonic jerking. Diagnosis was confirmed by 3 means: (1) Electroencephalogram demonstrating periodic sharp wave complexes, (2) MRI brain showing cortical ribboning and high signal in the caudate nucleus, and (3) presence of protein S100 and protein14-3-3 in the cerebrospinal fluid. Postmortem brain histology confirmed a typical spongiform encephalopathy. Establishing an underlying aetiology is dementia is important not only for prognostic reasons but in order to detect potentially reversible causes. In cases of an atypical dementing illness our proposed investigations may assist in confirming or excluding underlying Creutzfeltd-Jakob disease.

## 1. Introduction

Cognitive decline is a frequent cause of presentation to primary or secondary care, especially in the elderly: one in twenty people over the age of 65 develops dementia. As in the following case, there is often a paucity of clues as to the underlying pathology, and while the diagnosis will usually prove to be either Alzheimer's disease, dementia with Lewy bodies, or vascular dementia, there may be clinical and laboratory features suggesting rarer alternatives.

## 2. Case History

A 71-year-old Caucasian female presented with a 3-month history of cognitive decline, particularly short-term memory loss followed by intermittent dull headaches, which worsened progressively over the week prior to admission. She had type 2 diabetes and mild renal insufficiency but had previously been self-caring and independently mobile. Initial examination revealed no focal neurological signs but her Abbreviated Mental Test Score (AMTS) was 6/10. The only abnormal blood results were ESR 53, creatinine 154 mM, and glucose 11.4 mM. CT brain revealed a small region of subtle low attenuation in the right corona radiata, consistent with an infarct. This was a recent event as CT brain performed by her GP as part of investigations for memory loss two months earlier was normal. She was transferred to a nearby stroke rehabilitation hospital but her conscious level declined progressively over the following two weeks. She became increasingly confused, agitated, and withdrawn. This was initially attributed to an underlying chest infection but she did not improve with antibiotics. She was therefore readmitted to our service for further investigation.

**Figure 1:**
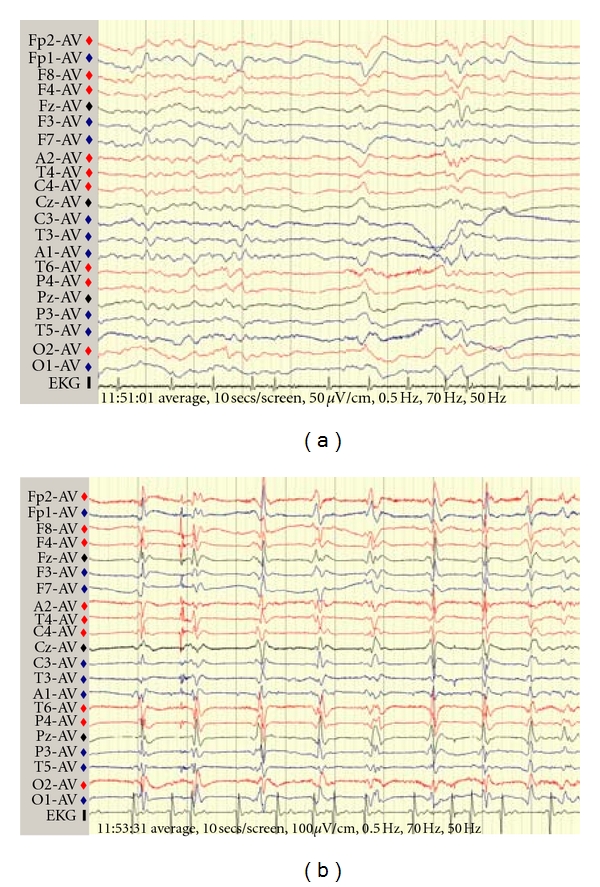
(a) Initial EEG demonstrating slow wave activity inconclusive for status epilepticus, (b) periodic sharp wave complexes demonstrated on EEG.

**Figure 2:**
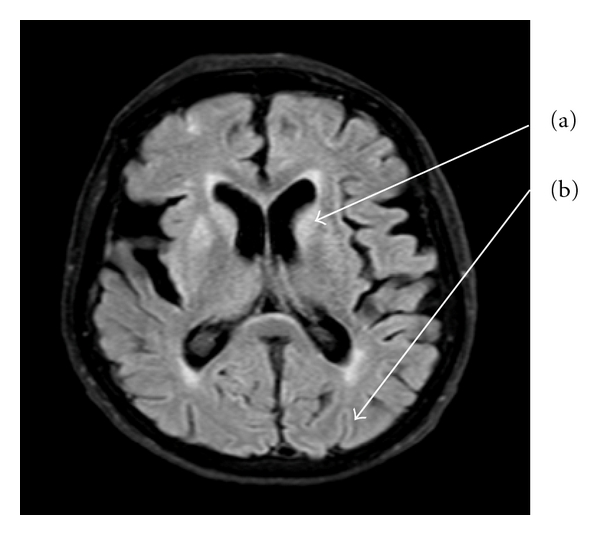
MRI scan demonstrating: (a) high signal in the caudate nucleus and (b) cortical ribboning.

**Figure 3:**
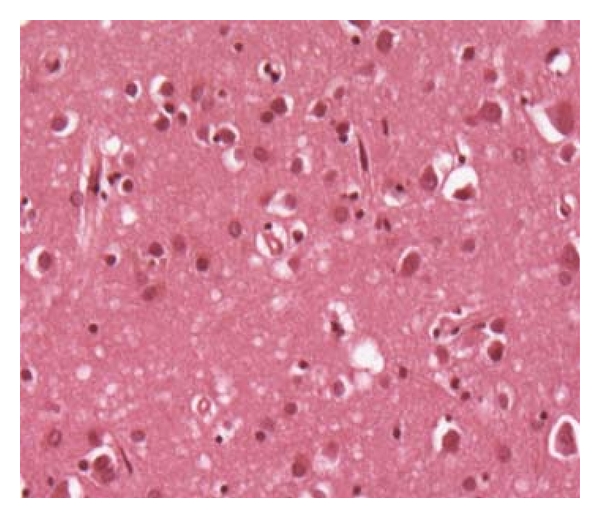
Spongiform encephalopathy (H&E stain).

On reassessment, 15 days after her initial presentation, she was apyrexial and haemodynamically stable but barely conscious with GCS of 8/15. Pupils were pinpoint, and she exhibited occasional myoclonic jerking of the limbs but no other focal neurology. EEG (Figure 1(a)) showed general slowing with some sharpened slow waves, sometimes followed by periods of suppressed EEG activity. This was not thought diagnostic of any specific entity but possibly consistent with a postepileptic state, and she was treated with phenytoin and later sodium valporate. Brain MRI brain showed the focal area of infarction in the deep white matter of right parietal regions, as noted previously on CT, together with cerebral atrophy, but no other abnormality. CSF was acellular, with normal protein, glucose and negative cytology, and negative HSV PCR. The syndrome of rapidly progressive cortical dysfunction with myoclonus without prominent focal neurological signs or major MRI brain scan abnormalities raised the possibility of CJD. CSF was sent to the CJD Surveillance Unit in Edinburgh (http://www.cjd.ed.ac.uk/) and revealed a significant increase in protein S100 to 1.0 (reference range <0.41). Protein “14-3-3” was also detected. At day 43 after initial presentation repeat EEG now showed periodic sharp waves followed by suppression, characteristic of CJD (Figure 1(b)). Repeat MRI revealed high signal in the caudate and basal ganglia, and ribbon-like cortical high signal change on the FLAIR sequence, also classic characteristics of CJD (Figure 2). Her conscious level failed to improve and the myoclonus progressed. She developed a hospital-acquired pneumonia and died seven weeks after the initial presentation. Postmortem examination of the brain demonstrated typical spongiform encephalopathy consistent with CJD. Neuronal loss and amyloid plaques were not mentioned in the report (Figure 3). There was no reference to the corona radiata vascular lesion. If further studies were to have been conducted, migration on Western blot analysis would have been helpful.

## 3. Discussion

CJD is a rare illness characterised by rapidly progressive dementia, ataxia, myoclonus, and akinetic, mutism [1]. At present the most reliable incidence figures come from Austria, where autopsy rates are over 90% for deaths in hospital. For the year 1995, an incidence of 1.38 per million was reported [2]. By far the most common form of CJD in elderly patients is sporadic CJD but cases of variant CJD have also been reported [3]. In all cases of CJD intracellular deposition of insoluble, abnormal form (PrPsc) of a normal soluble cellular protein (PrPc) in neurones is observed [4]. The disease has a particular predilection for the basal ganglia but neurones of the cerebral cortex and cerebellum are also commonly affected, leading to dementia, progressive akinesia, ataxia, and forms of epilepsy, notably myoclonus.

A large number of alternative diagnoses are possible when CJD is suspected, including rapidly progressive Alzheimer's disease, vascular dementia, and dementia with Lewy bodies [2]. However, myoclonus, ataxia, akinetic mutism, and visual hallucinations are significantly more common in CJD. Seizures ranging from simple seizures to status epilepticus, as in our case, have also been reported in CJD. Sporadic CJD patients have been misdiagnosed with non-convulsive status epilepticus (NCSE), highlighting that CJD should be considered a differential diagnosis in patients with status epilepticus [5, 6]. Generally, Alzheimer's disease is slowly progressive with a duration of 8–10 years, and neurological signs appear in advanced disease. Vascular dementia usually has a stepwise deterioration in cognitive function and can be associated with focal signs. Parkinsonism, visual hallucinations, and fluctuations in cognitive impairment are the main characteristics of dementia with Lewy bodies [4]. Other inflammatory conditions such as Hashimoto's encephalitis, cerebral vasculitis, neoplasia, storage diseases, and granulomatous disease must also be considered [6].

CJD progresses to death after a median illness duration of 5 months [7]. The median age of disease onset is generally lower for CJD patients than for Alzheimer's disease (66 years versus 71 years). Vascular dementia and dementia with Lewy bodies both have a similar age of onset of 68 years [4].

The World Health Organisation criteria for the diagnosis of sporadic CJD classifies possible CJD as rapidly progressive dementia of less than a two-year duration, with 2 of either (a) myoclonus (b) visual or cerebellar problems (c) pyramidal or extrapyramidal features or (d) akinetic mutism. Probable CJD must meet the criteria for possible CJD plus either a typical EEG or positive 14-3-3 in the CSF. CJD can only be classified as “definite CJD” once it has been neuropathologically confirmed.

## 4. Electroencephalogram

EEG studies can be a useful diagnostic aid in CJD. The characteristic feature is periodic sharp wave complexes (see Figure 1(b)). Although not pathognomonic, they are present in 52%–58% of patients with CJD but only around 6% of cases of Alzheimer's, Lewy body, and vascular dementia [4, 7]. EEG is also useful for ruling out nonconvulsive status epilepticus.

## 5. Magnetic Resonance Imaging

The most consistent finding on MRI is bilateral areas of hyperintensity in the caudate nuclei and putamina, with recent studies quoting specificity of up to 93% (see Figure 2). Hyperintensity is also seen to a lesser extent in the cortex, globus pallidus, and thalamus [8]. Some studies have proposed to amend clinical diagnostic criteria to include MRI findings, which showed to be 83% specific in one study [9]. Signal intensity changes in the cerebral cortex, called “cortical ribboning,” can also be seen but are less frequent. The degree of atrophy of the cortex and basal ganglia correlates with the disease duration [1]. Thus underlying the potentially important role in premortem diagnosis of sporadic CJD.

## 6. Cerebrospinal Fluid

CSF examination is imperative as a raised CSF cell count is the best single marker for a potentially treatable inflammatory process [6]. More detailed assessment for the presence of protein 14-3-3 is the most valuable factor in differentiating CJD and Alzheimer's disease. The presence and persistence of 14-3-3 is indicative of the more rapid brain destruction in CJD. It has a high sensitivity for sporadic CJD in the range of 88% to 97% and a specificity of 92% [4, 7]. Protein S100 is slightly inferior to 14-3-3 with an average sensitivity of 87% [10].

## 7. Brain Biopsy

Brain biopsy is generally regarded as a procedure of last resort due to perceived low diagnostic yield and possibility of serious complications including haemorrhage, infection, and seizures [5]. However, a study of 90 brain biopsies undertaken between 1989 and 2003 for the investigation of dementia found that 57% were diagnostic, the most frequent diagnosis being Alzheimer's disease (18%) while CJD was also frequently found, at 12%. A potentially reversible inflammatory cause was identified in 10% of cases [6]. Brain biopsy can therefore resolve diagnostic uncertainly but around 10% of people can expect to experience a complication and it is therefore generally reserved for younger patients, where there is a greater possibility of finding a reversible condition.

## 8. Summary

It is likely that CJD is underdiagnosed. In patients with rapidly progressive dementia we would recommend serial brain MRI and EEG examinations, and in particular, CSF examination for protein 14-3-3 as the three principal investigations. Although CJD is currently a fatal disease, establishing this diagnosis is not only essential in order to and exclude other treatable causes but also to be able to give patients and relatives accurate information about their diagnosis and prognosis.
